# Initial Application of Diffusional Kurtosis Imaging in Evaluating Brain Development of Healthy Preterm Infants

**DOI:** 10.1371/journal.pone.0154146

**Published:** 2016-04-21

**Authors:** Jingjing Shi, Liwen Chang, Jian Wang, Shun Zhang, Yihao Yao, Shuixia Zhang, Rifeng Jiang, Linying Guo, Hanxiong Guan, Wenzhen Zhu

**Affiliations:** 1 Department of Radiology, Tongji Hospital, Tongji Medical College, Huazhong University of Science and Technology, Wuhan, China; 2 Department ofneonatology, Tongji Hospital, Tongji Medical College, Huazhong University of Science and Technology, Wuhan, China; University of Pennsylvania, UNITED STATES

## Abstract

**Objective:**

To explore the parametric characteristics of diffusional kurtosis imaging (DKI) in the brain development of healthy preterm infants.

**Materials and Methods:**

Conventional magnetic resonance imaging (MRI) and DKI were performed in 35 preterm (29 to 36 weeks gestational age [GA]; scanned at 33 to 44 weeks postmenstrual age [PMA]) and 10 term infants (37.4 to 40.7 weeks GA; scanned at 38.3 to 42.9 weeks PMA). Fractional anisotropy (FA), mean diffusivity (MD) and mean kurtosis (MK) values from 8 regions of interest, including both white matter (WM) and gray matter (GM), were obtained.

**Results:**

MK and FA values were positively correlated with PMA in most selected WM regions, such as the posterior limbs of the internal capsule (PLIC) and the splenium of the corpus callosum (SCC). The positive correlation between MK value and PMA in the deep GM region was higher than that between FA and PMA. The MK value gradually decreased from the PLIC to the cerebral lobe. In addition, DKI parameters exhibited subtle differences in the parietal WM between the preterm and term control groups.

**Conclusions:**

MK may serve as a more reliable imaging marker of the normal myelination process and provide a more robust characterization of deep GM maturation.

## Introduction

Brain water content reduction and myelin maturation are the most important changes in the brain development of infants. Those changes can be visually evaluated on conventional magnetic resonance imaging (MRI) and quantitatively assessed using an advanced MR sequence. As a non-invasive and sensitive imaging modality, MRI has been widely used in the assessment of neonatal brain development[1–12].

Diffusion tensor imaging (DTI), an MRI technique based on the Gaussian diffusion hypothesis and diffusion tensor reconstruction, can provide information concerning the underlying microstructural characteristics of biological tissues by measuring the diffusivity of water molecules. Brain development is a complicated, long-term process that exhibits rapid changes between the third trimester of gestation and the first postnatal month[13–14]. Many studies have investigated this chronological development and the regional variations in brain maturation based on fractional anisotropy (FA) and apparent diffusion coefficient (ADC) values derived from DTI. The results have showed that FA decreases and ADC increases during the developmental process, particularly in white matter (WM)[3–4,8,14–20]. Recently, an advanced MR technique, diffusional kurtosis imaging (DKI), was introduced to characterize non-Gaussian water diffusion and tissue heterogeneity[21–26] by measuring the mean kurtosis (MK) value within a voxel across different cellular compartments and providing more accurate parameterization compared with DTI[27]. Previous studies[28–31]have demonstrated that DKI is useful for investigating ischemic stroke and neuropathologies such as Alzheimer’s disease and epilepsy. One study[32]demonstrated the advantages of DKI in assessing normal brain development in children ranging in age from newborn to 4 years old, but few studies have focused on neonatal brain maturation, particularly in the premature newborn brain. In this study, we aimed to explore the feasibility and application value of DKI in assessing the brain development of healthy preterm infants.

## Materials and Methods

### Subjects

This study was approved by the ethics committee of Tongji hospital, Tongji Medical College, Huazhong University of Science & Technology, and written informed parental consent was obtained for each infant prior to examination. Thirty-five preterm infants and ten term infants (control group) were enrolled between November 2011 and December 2013. The infants were sedated using oral chloral hydrate or enemas (20–30 mg/kg) and underwent both conventional MRI and DKI using a3.0T MRI scanner (GE Healthcare, Signa HDxt) with an 8-channel head coil. Neonatal earmuffs were used for hearing protection, and possible motion artifacts were mitigated by immobilization with a cotton pillow. An experienced neonatologist and a neuroradiologist were in attendance throughout the imaging process.

All selected infants met the following clinical criteria: (1) 1-min and 5-min Apgar scores>7; (2) no evidence of postanoxic encephalopathy, congenital infection or congenital anomaly syndrome; and (3) normal physical and neurological examination at 6 or 12 months PMA, as assessed by a board-certificated neonatologist. Additionally, all infants met the following radiological imaging criteria: (1) normal appearance on conventional MRI and (2) no obvious motion artifacts or an incomplete imaging process. PMA was estimated as GA at birth plus postnatal age at the time of the MR examination. The GA and PMA range of the preterm infants and term controls are presented in **S1 Fig.**

As shown in **S1 Fig**, we first divided the 35 preterm infants into two groups according to PMA at the time of MR scanning: the preterm infants before term-equivalent age group (less than 37 weeks; range, 33 to 36 weeks; 12 infants) and the preterm infants at term-equivalent age group (TEA group; 37 weeks or older; range, 37 to 44 weeks; 23 infants). Based on their GAs, the preterm infants in the TEA group were then divided into two subgroups: early preterm infant group (GA ≤ 32 weeks, 10 infants) and late preterm infant group (GA > 32 weeks, 13 infants).

### MR Acquisition and Image Analysis

The MRI protocols included fast recovery spin echo (FSE) T1-weighted imaging (T1WI; TR/TE = 360/11.4 ms); axial T2-weighted imaging (T2WI; TR/TE = 4260/102 ms); and a DKI series. For the DKI sequence, the diffusion directions = 25, and the b-values = 0, 1250, and 2500 s/mm^2^. Both b = 1250 s/mm^2^ and 2500 s/mm^2^ were included in one sequence, and two b0 were acquired in this sequence. The TR/TE was 7000/113 ms for both b = 1250 s/mm^2^ and b = 2500 s/mm^2^in this DKI sequence. The acquisition matrix was 128×130. The reconstruction matrix was 256×256. The acquisition resolution of the diffusion-weighted images was 1.4mmx1.4mmx4mm.The slice thickness was 4 mm without gap, the number of slices acquired in the DKI sequence was 18–24, the number of excitations (NEX) was 1, and the field of view (FOV) was 180 mm×180 mm. The total DKI scan time was 6 min 11 s.

**Fig 1:**
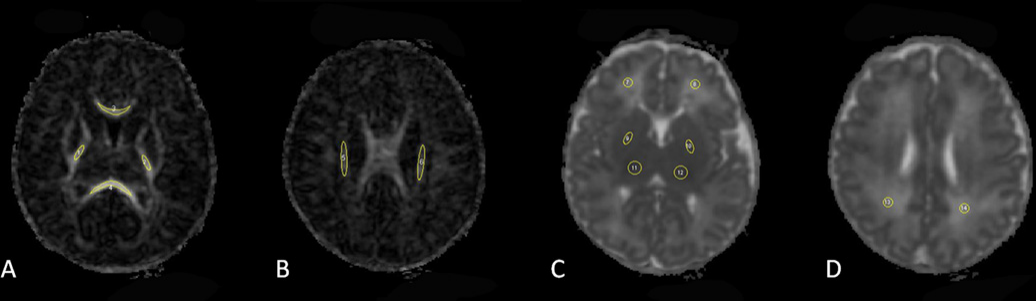
Position of ROIs on the FA (A, B) and MD (C, D) maps. A. Posterior limbs of the internal capsule (PLIC) and genu and splenium of the corpus callosum (GCC, SCC), which are labeled as 1–4, respectively. B. Corona radiata (CR), labeled as 5–6. C. Frontal white matter (FWM), lenticular nucleus (LN) and thalamus (TH), labeled as 7–12. D. Parietal white matter (PWM), labeled as 13–14.

All raw DKI data were processed using software developed by Tabesh[33] (Diffusional Kurtosis Estimator, version 2.0, http://academicdepartments.musc.edu/cbi/dki/DKE/dke_download.htm). Voxel-by-voxel analysis was performed after the images (b = 1250 and2500s/mm^2^) were registered to the b0 images using SPM8 and a nonlinear fitting algorithm (DKI fitting). The following parametric maps were then generated: MK, FA and mean diffusivity (MD). Regions of interest (ROIs) were manually drawn on transverse slices using ImageJ software (http://rsb.info.nih.gov/ij/) on the FA or MD map and then automatically projected onto the other two parametric maps. Eight different anatomical WM and GM structures were investigated (**Fig 1**), including the posterior limbs of the internal capsule (PLIC);the genu and splenium of the corpus callosum (GCC and SCC, respectively);the corona radiata (CR);the frontal and parietal WM (FWM and PWM, respectively);the lentiform nucleus (LN); and the thalamus (TH). Measurements were obtained by an experienced neuroradiologist who was blinded to the clinical information. The ROI size for each subject was identical for the left and right hemispheres. ROIs were placed at the center of structure of WM and deep GM areas to minimize variation. To evaluate intra-observer reliability, the neuroradiologist performed the same measurements for 15 randomly selected subjects (10 preterm and 5 term infants) 8 weeks later to avoid recall bias. To evaluate inter-observer reliability, another neuroradiologist who was also blinded to the clinical information performed the same measurements for the same 15 neonates.

### Statistical Analysis

The statistical analysis was performed using SPSS software (SPSS for Windows 18.0, Chicago, Ill), and *P*-valueless than 0.05 indicated statistical significance. Differences in clinical characteristics between groups were compared using Student’s t-test and chi-square test. Intra-class correlation coefficients (ICC) were calculated to assess intra- and inter-observer reliabilities of DKI measurements. The relationships among DKI-derived parameters (FA, MD and MK values) and PMA were analyzed using Pearson’s correlation analysis. The regional developmental differences of the selected WM regions were compared using the randomized block Student-Newman-Keuls test. The differences among the early preterm at TEA group, the late preterm at TEA group and the control group (term infants) were analyzed using one-way analysis of variance (ANOVA) with Bonferroni-type false discovery rate correction.

## Results

### Agreement Analysis

No significant differences were found in gender, PMA, or weight at the time of scanning, as shown in **Table 1**. The DKI-derived parameters showed good inter-observer and excellent intra-observer reliabilities for all selected ROIs (using the PLIC, SCC, PWM and LN as examples in **Table 2**).

**Table 1:** General demographics of infants.

Group	Preterm infants group with PMA at scan <37 weeks (n = 12)	Preterm infants group with PMA at scan≥37 weeks		Term born (n = 10)
		Early preterm (n = 10)	Late preterm (n = 13)	
M/F	8/4	9/1	10/3	9/1
Apgar (5min)	7.58	7.7	8.2	8.6
Mean GA (range; week)	32.0±2.0(29.0–35.1)	30.1±1.7(27.4–32.0)	34.7±1.7(32.6–36.9)	39.1±1.3(37.4–40.7)
Mean PMA at MRI (range; week)	34.9±1.2(33.0–36.3)	40.3±2.4(37.0–43.3)	39.7±2.1(37.6–44.0)	40.5±1.7(38.3–42.9)
Mean birth weight (range; kg)	1.75±0.3(1.20–2.26)	1.45±0.4(1.00–2.06)	2.12±0.4(1.60–2.87)	3.13±0.5(2.13–3.80)
Mean body weight at MRI(range; kg)	2.42±0.4(2.00–3.40)	3.07±0.4(2.30–3.40)	3.11±0.3(2.50–3.50)	3.42±0.4(2.70–4.00)

M/F = number of male and female infants; Apgar (5min) = Apgar score at 5 min; GA = gestational age; PMA = postmenstrual age. There were no significant differences in gender among these four groups (*P*> 0.05). The Apgar score in the preterm infant group before TEA (n = 12) and in the early preterm infant group at TEA (n = 10) were slightly lower than that in the term infant group (*P*< 0.05); there were no significant differences among these three preterm groups and between the late preterm infant group at TEA (n = 13) and the term controls (*P*> 0.05). The mean GA, mean birth weight, mean PMA and body weight at MRI of the preterm infant group before TEA (n = 12) were lower than those of the other three groups(*P*< 0.05).Mean GA and mean birth weight in the preterm groups at TEA were lower than those in the term control group (*P*< 0.05).There were no significant differences in mean PMA or body weight at MRI among the early preterm infant group at TEA, the late preterm infant group at TEA and the term controls (*P*> 0.05).

**Table 2:** Intra-observer and inter-observer variability of measurements.

ROI	Parameters	Intraclass correlation coefficient, 95% CI	
		Intra-observer	Inter-observer
PLIC	MK	0.953, 0.859–0.984	0.938,0.816–0.979
	FA	0.925, 0.777–0.975	0.885,0.657–0.961
	MD	0.961, 0.883–0.987	0.914,0.744–0.971
PWM	MK	0.926, 0.780–0.975	0.800,0.405–0.933
	FA	0.885, 0.657–0.961	0.738,0.219–0.912
	MD	0.911, 0.734–0.970	0.872,0.618–0.957
SCC	MK	0.936, 0.809–0.978	0.851,0.557–0.950
	FA	0.914, 0.743–0.971	0.876,0.631–0.958
	MD	0.899, 0.699–0.966	0.747,0.247–0.915
LN	MK	0.901,0.704–0.967	0.822,0.471–0.940
	FA	0.828,0.486–0.942	0.796,0.394–0.932
	MD	0.973,0.919–0.991	0.955,0.866–0.985

95% CI = 95% confidence interval; ROI = region of interest; PLIC = posterior limbs of the internal capsule; PWM = parietal white matter; SCC = splenium of the corpus callosum; LN = lentiform nucleus.

### Regular Developmental Pattern of the Preterm Infants

**Fig 2:**
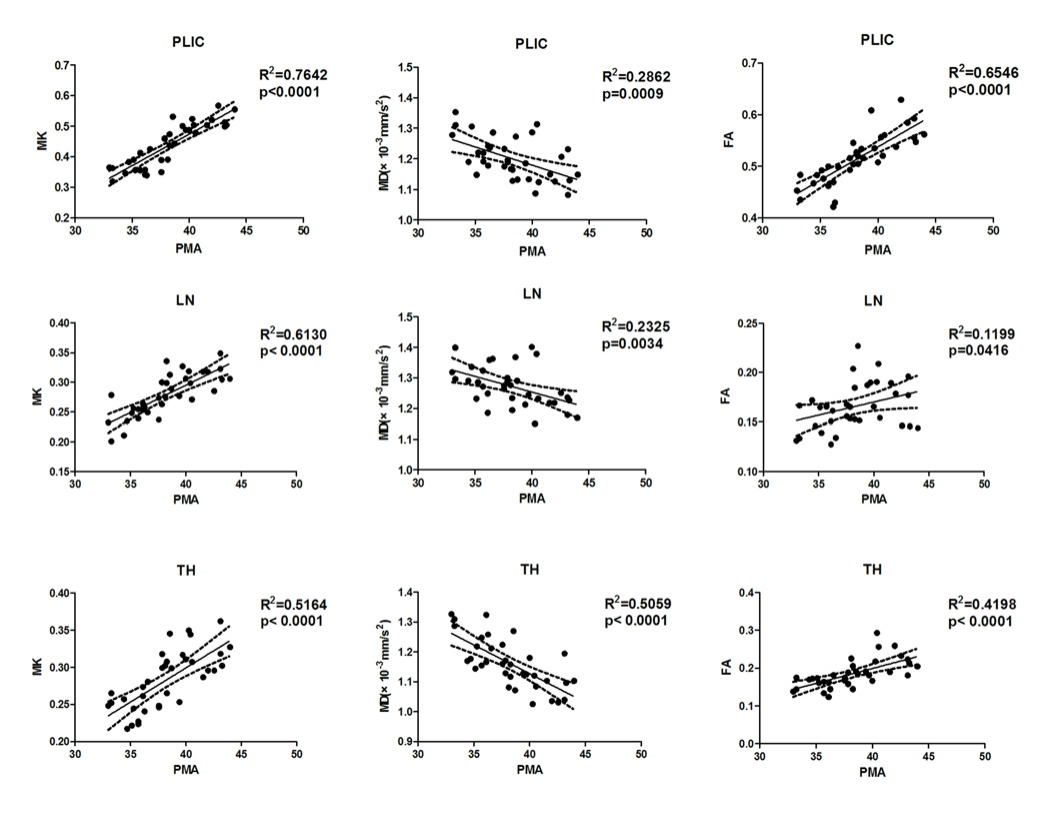
Correlations between PMA and quantitative DKI parameters in preterm infants.

As shown in **Table 3 and Fig 2**, the MK value was positively correlated with PMA in the projection and commissural pathways such as the PLIC, CR, GCC and SCC. In the WM areas, the best correlation between the MK value and PMA was observed in the PLIC(r = 0.874, P < 0.001). A positive correlation between the MK value and PMA was also found in the LN and TH (r = 0.783 and 0.719, respectively; P < 0.001). No significant correlations were found between MK values and PMA in the FWM (r = -0.268, P = 0.119) or PWM (r = -0.009, P = 0.957).

**Table 3:** Correlation coefficients between DKI values and PMA for all selected ROIs.

ROIs	preterm infants(n = 35)			term infants(n = 10)		
	FA	MD	MK	FA	MD	MK
White mater areas						
PLIC	0.809^**^	-0.535^**^	0.874^**^	0.695^*^	-0.128	0.879^**^
CR	0.497^**^	-0.587^**^	0.771^**^	0.221	-0.288	0.612
GCC	0.356^*^	-0.394^*^	0.602^**^	0.135	-0.440	0.566
SCC	0.674^**^	-0.296	0.607^**^	0.177	0.066	0.201
FWM	0.518^**^	-0.378^*^	-0.268	0.348	-0.337	0.026
PWM	0.586^**^	-0.480^**^	-0.009	0.573	-0.425	0.510
Grey matter areas						
LN	0.346^*^	-0.482^**^	0.783^**^	0.665^*^	-0.246	0.756^*^
TH	0.648^**^	-0.711^**^	0.719^**^	0.575	-0.228	0.717^*^

PLIC = posterior limbs of the internal capsule; CR = corona radiata; GCC = genu of the corpus callosum; SCC = splenium of the corpus callosum; FWM = frontal white matter; PWM = parietal white matter; LN = lentiform nucleus; TH = thalamus.

* indicates P < 0.05

** indicates P < 0.01.

The correlation between FA values and PMA was very similar to that of MK values and PMA in the WM areas. The FA value was strongly correlated with PMA in the PLIC, SCC and TH (r = 0. 809, 0.674 and 0.648, respectively) and moderately correlated in the CR, GCC, FWM, PWM and LN (r = 0.346 to 0.586).

MD values were strongly negatively correlated with PMA in the TH(r = -0.711) and moderately negatively correlated with PMA in the PLIC, CR, GCC, FWM, PWM and LN (r = 0.378 to 0.587). No significant correlation was found between the MD value and PMA in the SCC (r = -0.296, P = 0.084).

**Fig 3:**
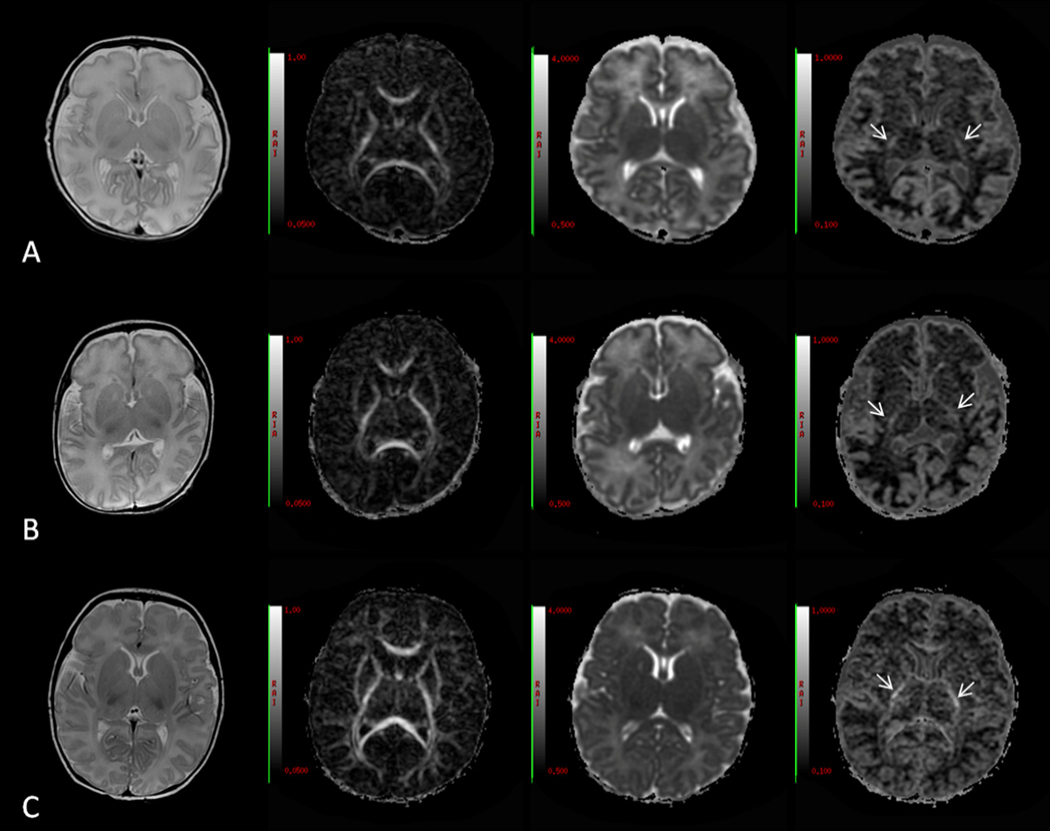
Conventional MRI and DKI of the preterm infants at different PMAs. Rows A to C represent PMAs of 34 weeks, 37 weeks and 40 weeks, respectively. The columns (left to right) represent the T2WI, FA, MD and MK maps. The RAI value range in the MD column was 0.5×10^-3^mm/s^2^~4×10^-3^mm/s^2^.The T2 signal intensity (SI) of the PLIC gradually decreased as PMA increased from 34 weeks to 40 weeks; meanwhile, the signal intensity on the FA and MK maps increased, as shown with a white arrow on the MK map. The image contrast between the GM and WM decreased on the T2WI and MD maps due to the decreased water content.

As shown in **Fig 3**, the T2 signal intensity (SI) of the PLIC gradually decreased when PMA increased from 34 weeks to 40 weeks; an increase in the corresponding FA and MK values was also observed. The tissue contrast between the GM and WM decreased on the T2WI and MD maps due to the decreased water content.

Similar developmental trends were observed in the term infants, but fewer regions had significant correlations compared to the preterm infants. The MK value was positively correlated with PMA in the PLIC, LN and TH (r = 0.879, 0.756 and 0.717, respectively; p < 0.05) in the term control group. The FA value was positively correlated with PMA in the PLIC and LN (r = 0.695 and 0.665, respectively; p < 0.05). No significant correlation was found the between these two values and PMA in the remaining ROIs.

### Developmental Differences in the WM Regions

Both the FA and MK values of the WM areas changed linearly with the increase in PMA in the preterm group, as shown above. Therefore, the randomized block Student-Newman-Keuls test was chosen rather than ANOVA to compare the regional developmental differences in each infant. As shown in **Table 4A and 4C**, the MK value was higher in the PLIC than in the SCC (0.437472 vs. 0.390371), followed by the CR, GCC, FWM, and PWM in decreasing order. The FA value was higher in the SCC than in the PLIC and GCC (0.605600 vs. 0.514835 and 0.514600, respectively), followed by the CR, FWM and PWM in decreasing order.

**Table 4:** Mean MK and FA values comparisons among different regions in WM areas.

**a**						
ROI	PWM	FWM	GCC	CR	SCC	PLIC
Mk_preterm_	0.205483	0.27613	0.324771	0.347587	0.390371	0.437472
**b**						
ROI	PWM	FWM	CR	GCC	SCC	PLIC
MK_term_	0.177691	0.24365	0.352944^*^	0.354^*^	0.4372^†^	0.458063^†^
**c**						
ROI	FWM	PWM	CR	GCC	PLIC	SCC
FA_preterm_	0.139647^△^	0.145171^△^	0.286381	0.5146^#^	0.514835^#^	0.6056
**d**						
ROI	FWM	PWM	CR	PLIC	GCC	SCC
FA_term_	0.162423^‡^	0.183193^‡^	0.290963	0.516736	0.5774	0.6297

Mk_preterm_(FA_preterm_) and MK_term_(FA_term_)refer to the MK values (FA values) in the preterm (n = 35) and term (n = 10)groups, respectively. The results show that the MK value increased gradually from the PWM to PLIC, while the FA value increased from the FWM and PWM to SCC. MK value was more consistent than the FA value in terms of myelination maturation, as previously described in a histological study[34].FA values between the FWM and PWM (^△^), and between the GCC and PLIC (^#^)in the preterm group did not show significant differences (*P* = 0.532 and 0.979, respectively). FA values between the FWM and PWM (^‡^) in the term control group did not show a significant difference (*P* = 0.277). MK values between the CR and GCC (*) and between the SCC and PLIC (^†^) in the term control group also did not show significant differences (*P* = 0.947 and 0.197, respectively). Differences among other values were statistically significant (*P*< 0.05).

In the term control group, the trend of developmental differences in the WM regions was consistent with that of the preterm group. As presented in **Table 4B and 4D**, the MK value was also higher in the PLIC than in the SCC, but the difference was not statistically significant. The FA values in the SCC and GCC were higher than the value in the PLIC.

### Subtle Differences Caused by Premature Delivery

**Fig 4:**
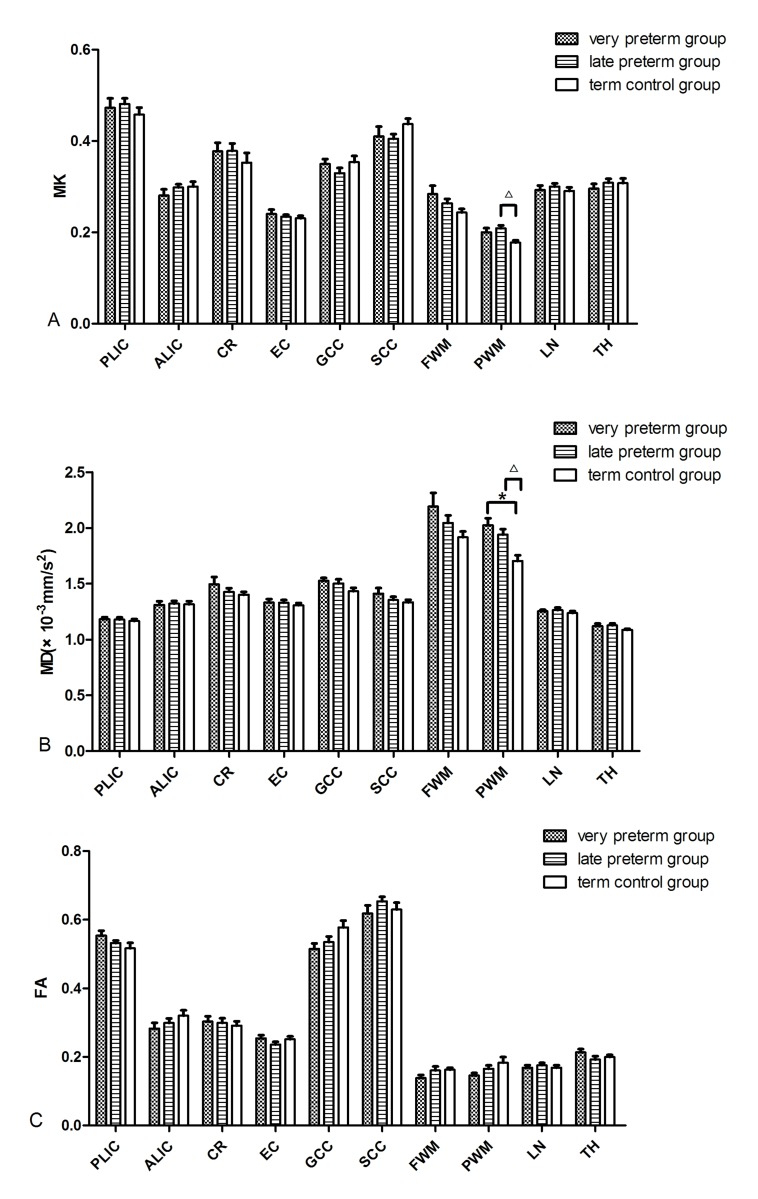
Representative bar graphs show the distribution of DKI-derived parameters for the anatomical WM regions among the three infant groups. Significant differences for the paired comparisons (*P*< 0.05) are denoted as follows: * indicates early preterm and term infants; **△** indicates late preterm and term infants.

As shown in **Table 5 and Fig 4**, significant differences were found among the three groups for MD and MK values in the PWM (F = 8.856, P = 0.001; F = 5.028, P = 0.013). Compared with the term control group, the MD values in the PWM for the early preterm infant and the late preterm infant groups were higher (1.70±0.16×10^-3^mm^2^/s vs. 2.03±0.20×10^-3^mm^2^/s and 1.94±0.17×10^-3^mm^2^/s, P = 0.001and 0.011, respectively). The MK value in the PWM of the late preterm infant group was higher than in the term control group (0.21±0.02vs.0.18±0.02, P = 0.012).No significant difference was found between the early and late preterm infant groups.

**Table 5:** Comparisons among the three groups with different gestational ages at the term-corrected age.

ROIs	Parameters	Early preterm group	Late preterm group	Term control group	*P*
PLIC	FA	0.55±0.04	0.53±0.03	0.52±0.05	0.127
	MD	1.18±0.06	1.18±0.07	1.17±0.05	0.862
	MK	0.47±0.07	0.48±0.04	0.46±0.05	0.583
CR	FA	0.30±0.05	0.30±0.05	0.29±0.04	0.854
	MD	1.50±0.21	1.43±0.11	1.40±0.08	0.319
	MK	0.38±0.06	0.38±0.06	0.35±0.07	0.555
GCC	FA	0.52±0.05	0.53±0.06	0.58±0.06	0.058
	MD	1.53±0.08	1.50±0.14	1.43±0.10	0.159
	MK	0.35±0.03	0.33±0.04	0.35±0.04	0.298
SCC	FA	0.62±0.08	0.65±0.05	0.63±0.06	0.387
	MD	1.41±0.15	1.35±0.10	1.33±0.07	0.295
	MK	0.41±0.07	0.40±0.04	0.44±0.04	0.268
FWM	FA	0.14±0.03	0.16±0.04	0.16±0.02	0.171
	MD	2.19±0.39	2.05±0.24	1.92±0.16	0.100
	MK	0.28±0.06	0.26±0.03	0.24±0.02	0.107
PWM	FA	0.15±0.02	0.17±0.04	0.18±0.05	0.107
	MD	2.02±0.20^*^	1.94±0.17^**△**^	1.70±0.16	0.001
	MK	0.20±0.03	0.21±0.02^**△**^	0.18±0.02	0.013
LN	FA	0.17±0.02	0.18±0.03	0.17±0.02	0.700
	MD	1.25±0.04	1.26±0.08	1.24±0.06	0.635
	MK	0.29±0.03	0.30±0.03	0.29±0.02	0.674
TH	FA	0.21±0.03	0.19±0.04	0.20±0.02	0.287
	MD	1.12±0.07	1.13±0.06	1.09±0.04	0.234
	MK	0.30±0.03	0.31±0.03	0.31±0.03	0.589

The unit of the MD value is10^-3^ mm^2^/s. Significant differences were found between the early preterm and term infants (*) as well as between the late preterm and normal term infants (**△**).

## Discussion

Our findings show that the brain maturation of preterm infants can be quantitatively evaluated with diffusional kurtosis imaging. The MK and FA values were comparable in the correlation with PMA in most selected WM regions. Specifically, the MK value had a greater positively correlated with PMA in the deep GM region compared with the FA value. These findings indicated that diffusional kurtosis imaging might provide more information on this complex developmental process.

The WM myelination process evolves in a spatiotemporal manner through the brain, roughly from central to peripheral, caudal to cranial and dorsal to ventral. Postmortem studies[34] have confirmed that mature myelination occurs earlier in the PLIC and CR, followed by the CC, and finally the lobar WM areas. Similarly, we found that the MK value decreased gradually from the PLIC to the cerebral lobe, which is in accordance with the order of the WM areas described above; these findings suggest the feasibility of using the MK value as a reliable indicator of the myelination process. The FA values reflected the same trend from central to peripheral. However, the FA value was highest in the SCC rather than in the more mature myelinated PLIC as we expected. A probable reason for this exception might be that the MK value is mainly determined by the extent of myelination, while the FA value is more strongly affected by closely packed fiber bundles. The fiber bundles appear to be more tightly packed in the SCC, leading to its higher FA value[20,32].For a relative comparison, we found that the FA values in the SCC and GCC were higher than the value in the PLIC in the term control group, which further confirms this point.

In the deep GM, the globus pallidus becomes myelinated early in the developmental process, and its dense structure may contribute to the increase in MK and FA values. In the thalamus, our result demonstrated that both the MK and FA values were better correlated to the PMA. This finding is consistent with previous studies[4,35]. Mukherjee et al. revealed that the maturational pattern of the thalamus was intermediate between the gray matter and the white matter structures. They indicated that its greater proportion of internal white matter tracts increased the FA value. That might explain our result that the thalamus had higher diffusion anisotropy than the lentiform nucleus.

DKI parameters exhibited subtle differences in the parietal WM between the preterm and term control groups. These differences are mainly attributed to the development delay caused by premature delivery. The proliferation of oligodendrocyte lineage precursors and increased intracellular compartments could account for the decrease in brain water content and the increase in membrane density [5,36]. The reduced MD value in the PWM might reflect a relatively lower water concentration because the premyelination process was slightly more advanced in the term controls. In addition, the MK value in the PWM of the late preterm infant group was higher than that of the term control group, which might indicated the presence of delayed underlying microstructural changes in the crossing fibers in the PWM. However, we found no difference in MK values in the PWM between early preterm infants at TEA and term infants. The PMA at the time of the scan should also be considered because earlier stimulation in the extrauterine environment could accelerate the development of white matter in early preterm infants compared to the late preterm infants[37].We also noticed similar developmental trends in the term infants but fewer regions exhibited significant correlations compared to preterm infants, perhaps also indicating that brain development was more accelerated in the preterm infants as compared to the term infants. Previous studies[38–39]have shown that very low birth weight preterm children had reduced FA values in the internal and external capsule; corpus callosum; and superior, middle superior and inferior fasciculus, and low FA values in these areas are associated with perceptual, cognitive, motor and mental health impairments. Longitudinal follow-up is needed to determine whether the underlying parietal changes are related to neurodevelopmental delays. In addition, Rose et al.[17]revealed significantly decreased FA and increased T2 values in the cerebral peduncle of term controls, which may indicate increased water content. Although our results demonstrated a decreasing trend of FA and MK values in the PLIC in the control group compared to the preterm group, no significant differences were found in these areas. This may be because the mean PMA of preterm infants at the time of scanning in Rose’s study was slightly higher than that of the term infants (41.1±1.1 vs. 39.8±1.6 weeks); however, there was no significant difference in mean PMA between the two groups in our study (40.3±2.4 vs. 40.5±1.7 weeks).The partial volume effect with the ROI method in our study might also contribute to the lack of significant differences in the group comparison of DKI measurements in the PLIC region.

There were several limitations in this study. First, the sample size of subjects was relatively small. Second, manual placement of the ROIs in the WM and GM regions might limit the reliability and reproducibility of the study. However, the ROI method is easy to implement and widely used in clinical practice, and the inter- and intra-observer reliabilities of these measurements were proven to be good to excellent. Third, as the histological validation study is warranted within this very early developmental age, the explanation for the meaning of DKI metrics with underlying biological changes is lacking.

## Conclusions

MK derived from DKI was proven to be more closely correlated with the extent of myelination progression shown in histological findings, which suggests the feasibility and potential application of the MK value as a reliable indicator during the myelination process. The MK value may provide a more robust characterization of deep GM maturation. In addition, DKI parameters exhibit subtle differences in the parietal WM between the preterm and term control groups, which may help with the understanding of early neurodevelopment.

## Supporting Information

S1 Fig Flow diagram of subject enrollment. (TIF)

## Abbreviations

MRI: magnetic resonance imaging
PLIC: posterior limbs of the internal capsule
GCC: genu of the corpus callosum
SCC: splenium of the corpus callosum
CR: corona radiata
FWM: frontal white matter
PWM: parietal white matter
LN: lentiform nucleus
TH: thalamus
DKI: diffusional kurtosis imaging
DTI: diffusion tensor imaging
FA: fractional anisotropy
MD: mean diffusivity
MK: mean kurtosis
GA: gestational age
PMA: postmenstrual age
