# Supplementary figures and images for: Initial Application of Diffusional Kurtosis Imaging in Evaluating Brain Development of Healthy Preterm Infants
Source: PLoS One. 2016 Apr 21;11(4):e0154146. doi: 10.1371/journal.pone.0154146 (PMC4839617; doi:10.1371/journal.pone.0154146)

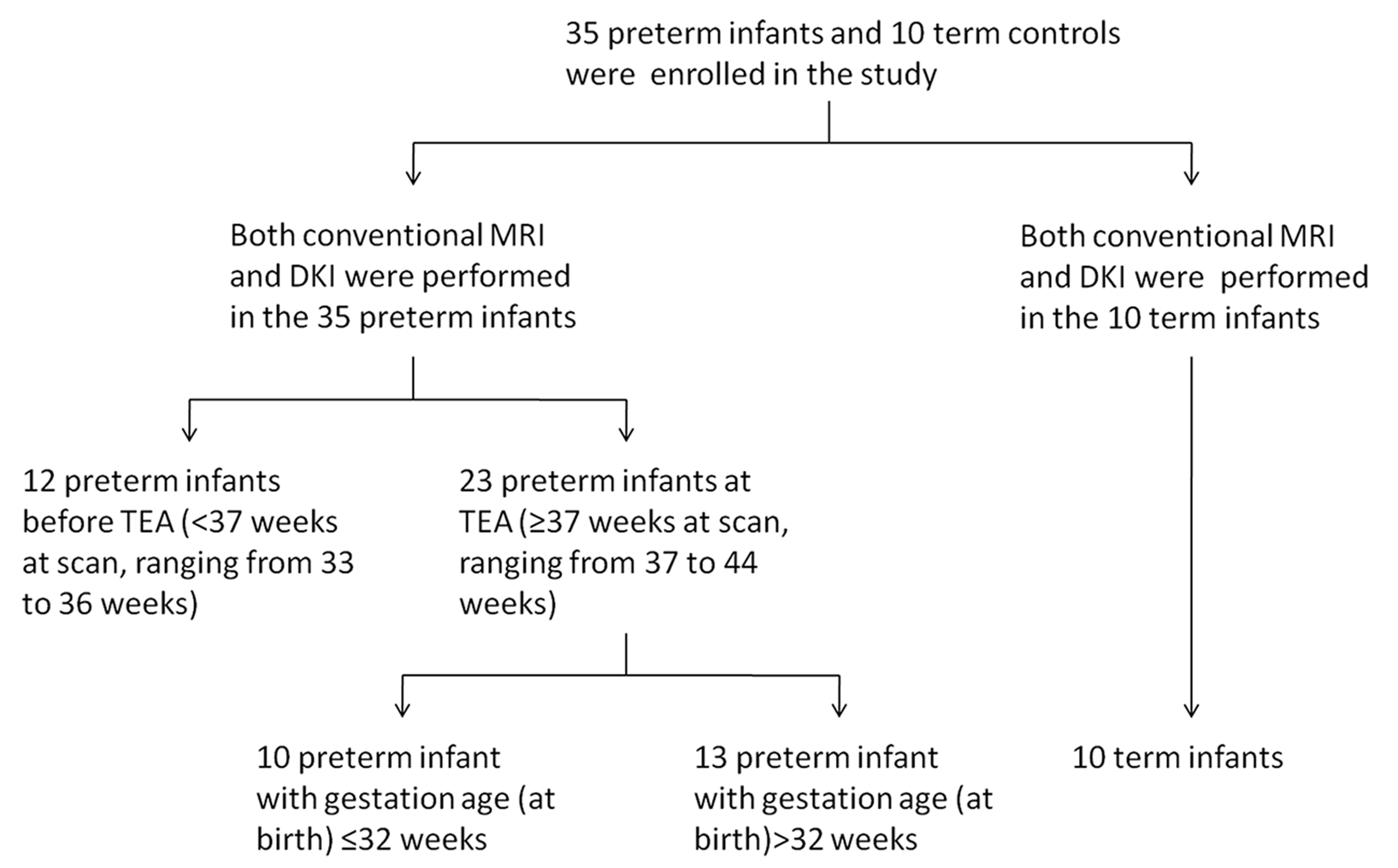

Supplement: S1 Fig — (TIF) [file pone.0154146.s001.tif]
